# Homozygous Loss of CDKN2 in Primary Cutaneous CD8(+) Lymphoma NOS

**DOI:** 10.1097/DAD.0000000000002613

**Published:** 2024-01-04

**Authors:** Alistair Robson, Joaninha Costa Rosa, Kristina Semkova, Farrah Bakr, Jose Cabecadas

**Affiliations:** * Instituto Português de Oncologia de Lisboa, Francisco Gentil, Lisboa, Portugal; and; † St John's Institute of Dermatology, Guy's and St Thomas' NHS Trust, London, United Kingdom.

**Keywords:** primary cutaneous peripheral T-cell lymphoma NOS, PTCL NOS, acral CD8^+^ lymphoma, aggressive epidermotropic CD8^+^ cytotoxic T-cell lymphoma, CDKN2, p16

## Abstract

Primary cutaneous acral CD8(+) lymphoma (AL) has been accepted as primary cutaneous acral CD8-positive T-cell lymphoproliferative disorder in the revised WHO and updated WHO-EORTC lymphoma classifications. Commonly arising on the ears and comprising a clonal cytotoxic CD8^+^ T-cell infiltrate, almost all cases follow an indolent clinical course. A single aggressive case reported in the literature had a deletion at the CDKN2 locus at 9p21. We report an atypical CD8^+^ T-cell proliferation arising on the chest of an elderly man who had some similarities to AL but with a very high proliferation rate, absent p16 protein expression, and homozygous loss of the CDKN2 locus using FISH analysis. A diagnosis of peripheral T-cell lymphoma not otherwise specified (PTCL NOS) was preferred. Analyses of 4 cases of AL demonstrated often low p16 protein expression but intact CDKN2 loci. This case raises the problems of the boundaries between AL and PTCL NOS, and a possible role in the loss of p16 function in pathogenesis.

## INTRODUCTION

Primary cutaneous acral CD8(+) lymphoma (AL) has provisional status in the revised WHO and updated WHO-EORTC lymphoma classifications.^1,2^ Most commonly arising on the ears as erythematous papules or nodules, almost all cases follow an indolent clinical course despite a diffuse infiltrate of clonal atypical cytotoxic CD8(+) T cells, often with the loss of 1 or more T-cell-associated antigens. A single case report detailed a patient with locally aggressive and multifocal disease, which invaded nasal cartilage and bone after a clinical course over 35 years. Genetic analysis demonstrated numerous abnormalities, including losses at the 9p21 locus.^3^

We encountered a solitary tumor of CD8^+^ peripheral T-cell lymphoma not otherwise specified (PTCL NOS) with some features resembling PCAL. In view of this, and the previous report of PCAL progression to an aggressive tumor, we assessed p16 immunoexpression and CDKN2A locus integrity using FISH analysis in this tumor and 4 cases of classical PCAL.

## CASES AND METHODS

**FIGURE 1.**
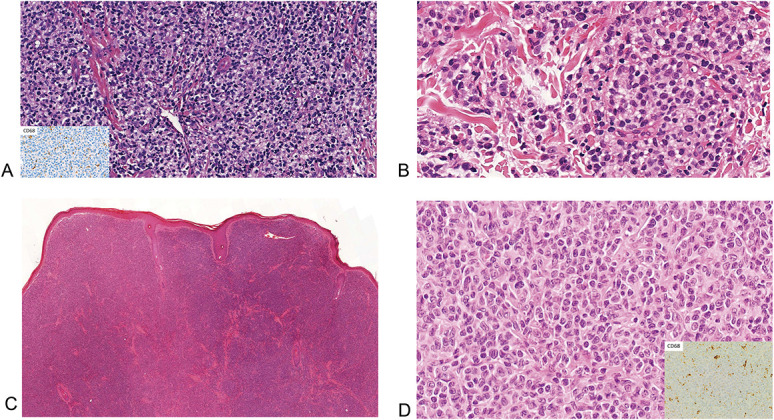
A, B, Diffuse dermal infiltrate of atypical mononuclear cells, with inset showing golgi-dot CD68 expression (cases 1 and 2). C, D, Diffuse nonepidermotropic infiltrate of atypical mononuclear cells (case 4), with inset of golgi-dot CD68 expression (A, H&E ×20, inset ×10; B, H&E ×40; C, H&E ×4; D, H&E ×40, inset ×10).

**FIGURE 2.**
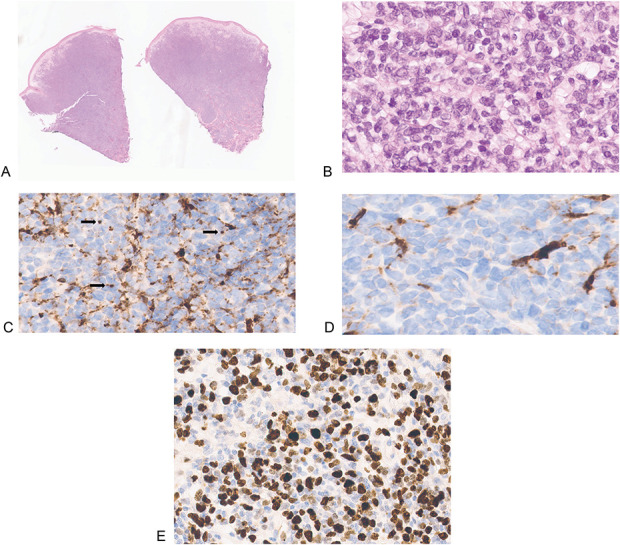
A, B, Diffuse nonepidermotropic infiltrate of atypical mononuclear cells, (C) CD68 dot-like expression (arrows), and (D) foci of CD68 negativity. E, Ki67 index is in excess of 50% (case 5). (A, H&E ×2; B, H&E ×100; C, ×100; D, ×150; E, ×100).

Five cases were included in the study. Four cases of primary cutaneous acral CD8(+) lymphoma were retrieved from the authors' archives. The clinical and histological features are listed in Table 1. Three of these (1–3) had been previously reported.^4,5^ (Figs. 1A, B). These cases were also subsequently immunostained for CD68, and each presented a dot-like pattern of positive expression. A further case (4) was a tumor arising on the ear of a 67-year-old man. Histopathology demonstrated a thin grenz zone with a dense, diffuse, dermal infiltrate of medium-sized atypical mononuclear cells (Figs. 1C, D). Immunohistochemistry demonstrated a CD8(+) TIA-1(+) CD99(+) T-cell phenotype with focal loss of CD2 expression and dot-like CD68 positivity (Fig. 1D inset); a T-cell clone was detected by PCR. The clinical, morphological, and immunophenotypic features are those of classical acral CD8(+) lymphoma. The final case (5) arose on the chest of a 71-year-old diabetic man, as three closely grouped nodules within a plaque that had developed over 1 year. Histologically, there was a nonepidermotropic, diffuse, dermal infiltrate of atypical small- to medium-sized mononuclear cells. Large cells/blasts were not apparent. Mitoses were readily identified (Figs. 2A, B). Immunohistochemistry demonstrated a CD8(+) TIA(+) Gr B (−) perforin(−) CD56(−) EBER(−) phenotype with CD68(+) dot-like foci; the latter were clearly identifiable but were not distributed throughout the tumor, having conspicuous areas of negativity (Figs. 2C, D). Ki67 suggested a proliferative fraction of around 60%-70% (Fig. 2E). TCR analysis revealed a dominant T-cell clone in a polyclonal background. Many of these features are in accord with acral CD8(+) lymphoma. CD68 dot-like positivity is a highly sensitive and specific marker for this entity,^6^ but this feature was only focally present, and the clinical appearance, location, and proliferative fraction are unusual. The absence of epidermotropism and preserved CD5 expression militated against a possible diagnosis of the rare solitary presentation of aggressive epidermotropic CD8^+^ lymphoma. Accordingly, a diagnosis of a CD8(+) T-cell lymphoma NOS, possibly developing from acral CD8(+) lymphoma, was preferred. The patient did not have “B symptoms” or lymphadenopathy and precautionary staging procedures, including a CT scan, failed to demonstrate extracutaneous disease.

**TABLE 1.** Clinical and Histopathological Features of Cases

Case	Age, yr/Sex	Location	Immunostaining (+)*	Morphology	p16
1†	68/M	Left ear helix single papule	Focal granzyme B expression Ki67 40%	Dense mononuclear cell infiltrate of atypical medium-sized cells with moderate pleomorphism. Mitoses identified. Focal epidermotropism with 2 Pautrier microabscesses	20%
2†	70/M	Left heel, single plaque	Loss of CD2 Ki67 20%	Dense, nonepidermotropic, atypical, moderately pleomorphic, dermal lymphoid infiltrate	5%
3†	47/F	Right dorsum of foot, papule; papules/nodules hand, nose, foot	Loss of CD2 and CD7 Ki67 20%–30%	A perivascular infiltrate of atypical mononuclear cells in the papillary and upper reticular dermis. No epidermotropism	5%
4	67/M	Right ear, single nodule	Partial loss of CD2 Ki67 20%	Dense diffuse infiltrate of atypical, small-to-medium, atypical, lymphoid cells. Grenz zone	<1%
5	71/M	Chest, 3 nodules	CD8(+) CD68(±) Ki67 60%–70%	Dense, nonepidermotropic, mononuclear infiltrate of moderately pleomorphic atypical cells. Mitoses	<1%

* Cases 1–5 were all diffusely CD8(+), with dot-like CD68(+), CD4(−), CD30(−) EBER(−), and had a T-cell clone.

† Previously reported.^4,5^

**FIGURE 3.**
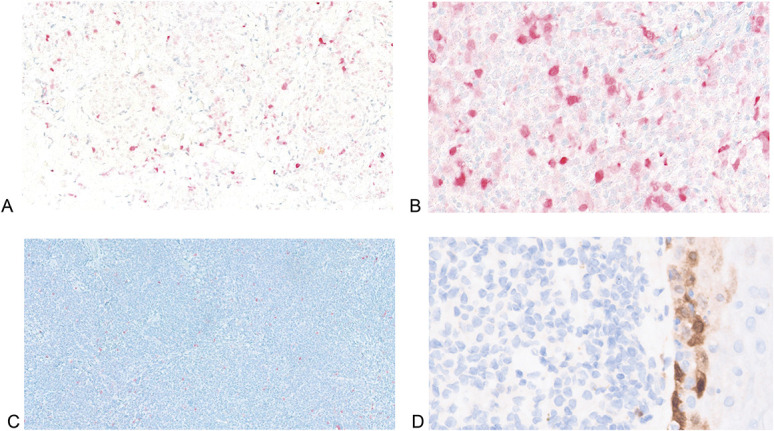
A, B, Low prevalence of p16 protein in cases 3 and 1. C, D, Complete absence of p16 immunoexpression (cases 4 and 5; epidermis to right in 5) (A, ×20; B, ×40; C, ×10; D, ×100).

**FIGURE 4.**
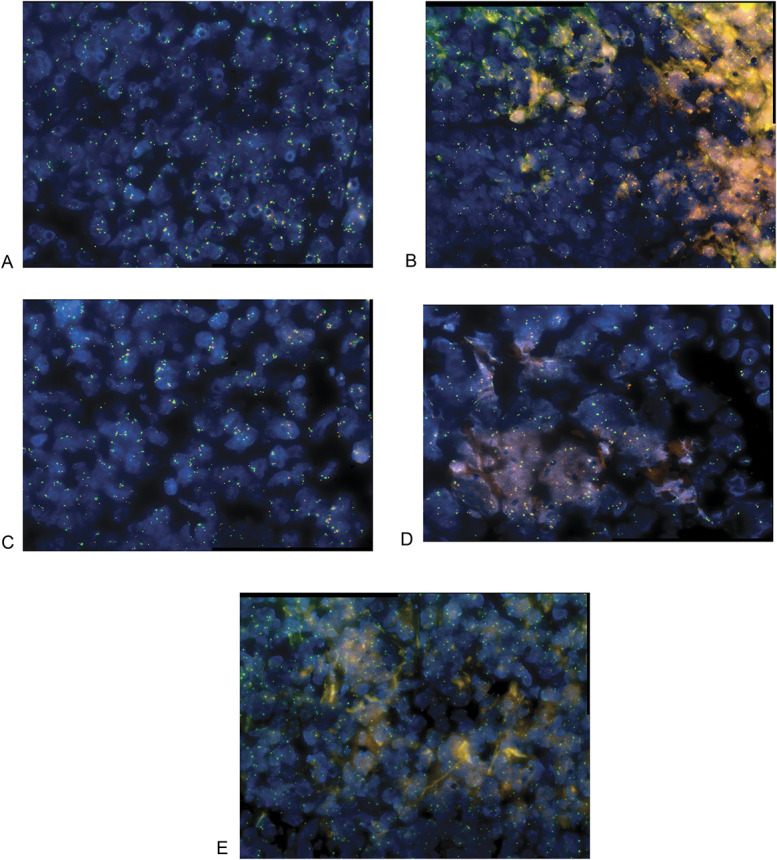
A–D, Fluorescence in situ hybridization in cases 1 to 4 showing no deletion of *p16/CDKN2A.* E, Case 5 with loss of red signals (*p16/CDKN2A* probe). Green (centromere of chromosome 9) to red (*p16/CDKN2A*) signals ratio is 1:1.

All 5 cases were analyzed by immunohistochemistry for p16 protein (Ventana anti-p16 INK4a (E6H4)). In 3 cases, there was protein expression, albeit by a minority of the neoplastic T-cell population, varying from 5% to 20% (Table 1, Figs. 3A, B). However, two cases, including the atypical tumor (4 and 5), were almost completely negative for p16 (<1% and 0) (Figs. 3C, D). All cases were subsequently analyzed by FISH for the CDKN2A locus at 9p21(Vysis CDKN2A/CEP 9 FISH Probe Kit by Abbott), which encodes the p16 protein. Four demonstrated a normal pattern, including case 4, which had <1% p16 immunopositivity, but an abnormal signal denoting loss of this locus was found in case 5, the atypical tumor (Fig. 4).

## DISCUSSION

Primary acral CD8(+) lymphoma was first recognized as a distinct entity in 2005 and originally termed indolent CD8(+) lymphoid proliferation of the ear.^7^ Subsequent reports confirmed an unusual and striking predilection for the ears but also documented tumors at other, preferentially acral, sites.^4,5,8–19^ However, rare examples at nonacral locations are described.^3,20^ The classic example has a reproducible morphology of a nonepidermotropic dense infiltrate of small- to medium-sized atypical cells and a constitutive cytotoxic T-cell signature, namely, CD8(+) TIA-1(+) but granzyme B (GrB)(−) perforin (−), usually CD99(+) and with the loss of 1 or more T-cell-associated antigens. The proliferation fraction is typically low, with few or no mitoses, and a Ki67 fraction of <20%.^4,5,7–19^ Nevertheless, otherwise, typical tumors may have 1 or several unusual pathological features without apparent clinical significance; these include focal epidermotropism and Pautrier microabscesses, cytotoxic activation markers, and higher proliferative fractions.^4,5,21,22^ Wobser et al and Kempf et al demonstrated a uniform, peculiar, dot-like pattern of CD68 expression by the neoplastic cells^6,23^ and suggested that this was a highly sensitive and specific feature of this tumor.

Although overwhelming evidence points to a benign clinical course for patients, Alberti Violetti et al^3^ reported a single case that followed an aggressive course albeit after 35 years. Genetic analysis demonstrated several abnormalities, including losses of 9p21.3-p21.1, 9q21.11 loci, which includes the coding region for the p16 protein, although protein expression by immunohistochemistry was not documented. This tumor demonstrated an initial proliferation fraction of 10%, which rose up to 60% in later samples.

The p16 protein acts to bind CDK (cyclin dependent kinase) 4 and CDK6, resulting in hypophosphorylation of retinoblastoma protein acting to inhibit cell cycle progression, effecting arrest at the G1/S checkpoint. A role for p16 inactivation is implicated in a wide variety of tumors, including melanoma, pancreatic and breast adenocarcinomas, squamous cell carcinoma of the head and neck, and mesotheliomas. The mechanisms of inactivation include point mutations, homozygous deletions, promoter hypermethylation, and loss of heterozygosity. Of these, homozygous deletions and hypermethylation are the most common and result in nonfunctional p16 protein.^24^ Abnormalities of p16 are well documented in a variety of leukemias and lymphomas, including T-cell lymphoblastic leukemia, non-Hodgkin lymphomas, including MALT type, CLL, and diffuse large B-cell lymphoma.^25–29^ Loss of 9p21 is well characterized in primary, cutaneous, diffuse, large B-cell lymphoma, leg type,^26^ and in cases of mycosis fungoides and Sezary syndrome.^30^ Interestingly, there is evidence that abnormalities of the p16 pathway are associated with the progression of disease and an aggressive biological course in both systemic and cutaneous B-cell and T-cell lymphomas and to be associated with shorter survival.^28,30^

All 4 typical cases of acral CD8(+) lymphoma in this study had low immunohistochemical expression of p16, indicating that this may be a consistent feature of this lymphoma. In 3 cases, this ranged from approximately 5%–20%. Although this suggests downregulation of the protein, and possible inactivation, the benign clinical course indicates that this is unlikely to have biologically significant consequences for tumor progression. By contrast, homozygous deletions of the 9p21 locus, which FISH detection of CDKN2 loss most often reflects, is a documented association with progression and aggression in diffuse, large, B-cell lymphoma and mycosis fungoides^28,30^; furthermore, Tsang et al^31^ reported a unique case of primary cutaneous follicle center cell lymphoma, of high-grade cytological morphology, with CDKN2A deletion that progressed to diffuse, large, B-cell lymphoma. The demonstration of 9p21 loss in the only aggressive case of acral CD8(+) lymphoma to date^3^ is in accord with this putative role in tumor progression; this tumor also had a high proliferation rate. The finding of the deletion in the CD8^+^ lymphoma NOS in case 5 in our series might indicate progression from previous acral CD8(+) T-cell lymphoma, and it may reflect an inherently more aggressive biology and a basis for the observed very high proliferation rate. Currently, classical examples of acral CD8(+) lymphoma do not require staging, and treatment options often involve simple excision or superficial radiotherapy.

Whether or when atypical cases of acral CD8^+^ lymphoma require staging and more active management is unclear, but we suggest that caution is warranted in these atypical examples and certainly staging investigations are justified, with complete tumor removal advisable. Finally, the near universal indolence of acral CD8(+) lymphoma has led to consideration to “downgrade” the name of the entity to “lymphoproliferative disease” as now used in CD4(+) pleomorphic lymphoproliferative disorder (CSMPTCL).^32^ However, unlike CSMPTCL, acral CD8(+) lymphoma is clearly a monotonous, clonal, cytologically malignant, neoplastic population and often occurs with T-cell antigen loss, which are collective cardinal features of lymphoma. Furthermore, it has been reported that the risk of recurrence in acral lymphoma rises with earlier age at presentation.^33^ The observation of rare examples with p16 deletions, and atypical or aggressive correlates, while exceptional, serves as an additional cautionary note to this proposed amendment to nomenclature.
